# Pulmonary vascular disease in Africa: Lessons from registries

**DOI:** 10.21542/gcsp.2020.2

**Published:** 2020-04-30

**Authors:** Ana Mocumbi, Adjine Mastala, Phath Guambe, Anastase Dzudie

**Affiliations:** 1 Instituto Nacional de Saúde, Marracuene, Mozambique; 2 Universidade Eduardo Mondlane, Maputo, Mozambique; 3 Faculty of Medicine, University of Yaoundé, Yaounde, Cameroon; 4 Douala General Hospital, Douala, Cameroon; 5 Clinical Research Education, Networking and Consultancy, Douala, Cameroon

## Abstract

The epidemiology of pulmonary vascular disease (PVD) remains unclear in Africa, where health systems do not reach the majority of the population and heath information systems are poorly developed. In this context, registries are particularly important in gathering crucial information on PVD, aiming at improving knowledge of the epidemiology and/or quality of care. While population-based registries are the main tool to identify incident cases, and be a better indicator of pulmonary vascular disease burden, hospital-based registries can give an indication of the demand for specific care services, which is useful for health policy and planning.

The only registry for pulmonary hypertension in Africa – the Pan African Pulmonary Hypertension Cohort (PAPUCO) – involved four countries, and was a pragmatic study that revealed a unique pattern of environmental risks, issues related to low access to health care, and ill-equipped health facilities for diagnosis and management of pulmonary hypertension. In addition, disease specific registries for conditions such as congenital heart disease and rheumatic heart disease uncovered high occurrence of PVD that can be managed and/or prevented with improvements in community awareness, surveillance, management and prevention.

It is suggested that existing networks of experts and researchers develop regional registries to determine the epidemiology of PVD in Africa, assess geographic, environmental and seasonal differentials, as well as inform policy and care provision in the continent.

## Background

Registries use observational methods to collect uniform data on individuals with a specific disease, and are of two major types: hospital-based and population-based. They serve as clinical information systems that identify relevant sub-populations for proactive care, facilitate individual patient care planning, allow sharing of information to coordinate care, and monitor performance of practice team and care system.

With progress in information and communication technologies, registries can be collected at national, regional and global levels, allowing better characterization of disease burden and profile, as well as raising attention to similarities and differences among geographically separated regions.

In areas where the health information systems are poorly developed, registries are particularly important in gathering of crucial information on specific conditions, aiming at improving knowledge of the epidemiology and/or quality of care. However, the establishment of effective population-based registries (PBRs) in low-resource settings is challenging. Access to health care in rural remote areas is low, lack of knowledge and awareness of common conditions among patients cause them not to look promptly for health care, and – in many countries – there is lack of accurate population censuses. Thus, hospital-based registries (HBRs) – more frequently used in these under-resourced areas – have a different and complementary role to PBRs, focusing mainly on clinical information about patients as well as on their outcomes. HBRs can provide data about the mode of diagnosis, the clinical features of the condition, use of therapeutic options, patient follow-up details, and can be a source of information for PBRs.

The diagnosis of pulmonary vascular disease (PVD) is technically demanding and definitions have been evolving^1^, partially explaining why the global epidemiology remains unclear. In Africa, where the health systems do not reach the majority of the population, PBRs would be the main tool to identify incident cases and be a better indicator of PVD burden. Alternatively, HBRs can give an indication of the demand for specific care services, which is useful for health policy and planning^2^.

However, due to limited local resources, low availability of accurate information regarding the number of patients affected, lack of expertise and equipment for accurate diagnosis, and low availability of accurate data about the population, information about PVD burden on the African continent is scarce. A search on the PubMed for registries in different countries in Africa carried out based on proper keywords in English – including “hospital-based”, “clinical” and “registry” and “pulmonary hypertension” – reveals only one hospital-based registry of pulmonary hypertension (PH)^3^, and there are no studies on its incidence.

## Historical perspective

The *Heart of SOWETO* study published in 2006 looked at the general profile of cardiovascular disease in an urban community in South Africa and described high occurrence of right heart failure (RHF) and PH^4^. This clinical registry captured data from 5328 *de novo* presentations of heart disease over two years (2006–2008), and detected 697 (28%) patients with RHF (50% primary diagnosis) among the 2505 cases of heart failure (47% of total cohort).

Among the many pathways to RHF the most common were:

i. concurrent left-sided heart disease (213 cases, 31%) such as hypertensive heart disease, dilated cardiomyopathy, peripartum cardiomyopathy and rheumatic heart valve disease
ii. chronic lung disease (179 cases, 26% including COPD and tuberculosis); and
iii. pulmonary arterial hypertension (PAH) (141 cases; 20%) – with women being almost two-fold more likely to present with PAH (OR 1.72, 95% CI [1.17–2.55]; P = 0.006)^4^.

In Mozambique, Mocumbi et al.^5^ retrospectively studied 534 patients with congenital heart disease assisted at a referral unit between 2001 and 2007, collecting epidemiological, clinical, echocardiographic and surgical data from hospital files. A pattern of late presentation was revealed - median age at diagnosis was 4 years – with high occurrence of complications, including fixed PAH in 45 (8.4%) patients out of the 481 with indication for surgery. More recently in Senegal, PH was found in 50% of patients (N=50) aged at least 16 years and followed for congenital heart disease in the cardiology department in Dakar between May 2003 and March 2015^6^.

Two registries of rheumatic heart disease show the relevance of preventable conditions as cause of PH in Africa; the diagnosis of PH was based on echocardiography.

First, the VALVAFRIC study^7^, a multicenter, hospital-based retrospective registry of patients with RHD hospitalized in 12 cardiology departments from seven African countries, having at least one mild RHD lesion seen on echocardiography between 2004 and 2008. Out of the 3441 patients included in the study, 1385 had severe lesions (803 women; mean age 29.3 ±15.6 years) and combined valvular lesions were observed in 13% of cases. PH was present in 28.7% patients and dilatation of the right cardiac chambers in 19.8%. The ratio of severe to any RHD valvular lesion was higher in countries with the lowest gross domestic product. Although 1200 patients required valve repair or replacement, only 27 had surgery.

The second registry – the Global Registry of Rheumatic Heart Disease^8^ – enrolled prospectively 3343 patients presenting between January 2010 and November 2012 to 25 centers in 12 African countries (apart from India and Yemen), who were then followed for two years to assess mortality and other adverse outcomes. Overall 3343 patients (median age 28 years; 66.2% female) were enrolled, the majority (63.9%) with moderate-to-severe multivalvular disease. PH was present in 28.8% patients at diagnosis, being the second commonest complication after congestive heart failure (33.4%)^8^.

**Table 1:** Summary of studies published between 2014 and 2019 with data on occurrence of pulmonary hypertension in Africa.

Author	Study Design, Number of Patients, Heart Condition and Age of Participants	% with PH
Ifeoluwa et al. 2019^10^	Case-Control; 79 hypertensive heart disease and 92 healthy controls (adults)	32.9
Lamina et al. 2019^11^	Case-Control; 200 sickle cell disease and 200 normal controls; patients 1-12 years old	8.0
Mahomed et al. 2019^12^	cross-sectional study; 228 COPD - adults	63.0
Bigna et al. 2019^13^	Systematic review; 42,642 HIV infected adolescents and adults from 17 countries	8.3
Kushimo et al. 2019^14^	Observational; 219 patients with heart failure - adults	8.8
Dzudie et al. 2018^15^	Prospective cohort study; 2194 patients adults	15.6
Bigna et al. 2017^16^	Systematic review of observational studies; 11,163 people w/ cardiac complains - adults	9.8
	937 HIV positive	10.6
	2077 heart failure	32.9
	259 undergoing heart surgery	68.7
	51 COPD	62.7
Jinji et al. 2017^17^	Cross-sectional; 178 adults	25.3
Sokunbi et al. 2017^18^	Case-Control; 175 sickle cell diseases; 5-18 years old	22.9
Amadi et al. 2017^19^	92 sickle cell disease –adults	23.9
Bigna et al.2016^20^	Systematic review and meta-analysis; 664 adult patients (3 studies)	14.0
Ejim et al. 2016^21^	259 patients w/ degenerative mitral valve disease	30.0
Adem et al. 2014^22^	Retrospective review of records; ultrasound department evaluations in adults	32.6
Enakpene et al. 2014^23^	90; patients w/sickle cell disease - adults	12.2

The utilization of valvuloplasty and valve surgery was low overall, being higher in upper-middle compared with lower-income countries. The vital status at 24 months was known for 2960 (88.5%) patients – again two-thirds were female; although patients were young (median age at death was 28.7 years) and the case fatality rate was high (500 deaths, 16.9%), representing a mortality rate at 116.3/1000 patient-years in the first year and 65.4/1000 patient-years in the second year^9^. Although PH was not among the independent predictors of death it surely contributed to higher morbidity and low quality of life.

The screening of the titles and abstracts of original research into PH published on PubMed in the last 5 years (2014–2019), found 14 publications that provide data on the occurrence of PH in several heart and lung conditions^10–23^, which varied between 8% among HIV infected individuals and sickle cell anemia, to as high as 68.7% in patients undergoing cardiac surgery (Table 1). Most studies used transthoracic cardiac ultrasound to estimate the pulmonary arterial pressure.

## The Pan African Pulmonary Hypertension Cohort (PAPUCO) study

The Pan African Pulmonary Hypertension Cohort (PAPUCO) research group was established in 2011 to create a prospective registry cohort study of *de novo* pulmonary hypertension (PH) cases in Africa.^3^ Recognizing that the use of medical records would jeopardize the collection of minimal data for diagnosis, and acknowledging the challenges of archiving medical records in some hospitals in the participating countries, the team decided to do a prospective registry allowing collection of detailed data from patients with definite diagnosis based on criteria agreed by all participants and subjected to quality control.

The multinational, multi-centre, registry-type cohort study was established and tailored to resource-constraint settings to describe disease presentation, disease severity and etiologies of PH, comorbidities, diagnostic and therapeutic management, and the natural course of PH in Africa^3^. PH was diagnosed by specialist cardiologists using echocardiography (right ventricular systolic pressure >35 mmHg, absence of pulmonary stenosis and acute right heart failure), usually accompanied by shortness of breath, fatigue, peripheral oedema and other cardiovascular symptoms, electrocardiogram and chest X-ray changes in keeping with PH as per European Society of Cardiology and European Respiratory Society (ESC/ERS) guidelines.

Complementary investigations such as computerized tomography (CT) scan, ventilation/perfusion scan or right heart catheterization were performed at the discretion of the treating physician and the site capacities. Functional tests included a 6 min walk test and the Karnofsky Performance Score. The WHO classification system for PH was applied to describe the different aetiologies of PH. All local ethics committees of the participating centres approved the protocol.

Consecutive patients from nine specialist care referral centers in Cameroon (109), Mozambique (35), Nigeria (33) and South Africa (43) were recruited over 24 months. A pragmatic approach to diagnosis was agreed, and the inclusion criteria were the following:

i. newly diagnosed with PH based on standardized clinical and echocardiography criteria;
ii. capacity to return for 6-month follow-up if alive.

Participating center’s eligibility was based on availability of echocardiography with experience of assessing of right heart function; experience in diagnosing PH according to World Health Organization (WHO) classification; experience in clinical management of patients with RHF; and availability of resources for 6-month follow-up^3^.

Two hundred–twenty patients were recruited; 209 were adults (median age 48 years), 97% of African descent and 124 (59%) women. Two-thirds presented in WHO FC III or IV; 1/3 could not walk further than 300 m on 6MWT; of those with 6-month follow-up data (N=189) 39 (21%) had died. Indoor cooking and/or heating was reported by 66 patients (32%), with predominance of women (52;44% vs 14;17% for men; p=0.0001).

Previous or concurrent pulmonary tuberculosis was reported by 47 (23%) - of which 10 patients (5%) had concurrent tuberculosis when included in the study. At the time of diagnosis 134 (64%) were subjected to active screening for HIV, revealing 47 patients with positive test (35%). Interestingly, despite 167 patients (80%) living in areas endemic for schistosomiasis only one patient was diagnosed with this condition.

An interesting aspect of multimorbidity was the finding of systemic hypertension and diabetes in 87 (42%) and 17 (8%) patients, respectively^3^ (Table 2). The 11 children recruited presented with dyspnoea, fatigue, cough, and palpitations; six children had concurrent PH associated congenital heart disease and three were diagnosed with left heart disease.

**Table 2:** Selected risk factors profile and clinical findings of 209 adults (85 female; median age 48 years) presenting with PH.

Characteristic	Number of Patients
**Risk Factors**	
African descent	203 (97%)
Primary education or less	125 (60%)
Systemic Hypertension	87 (42%)
Chronic Obstructive Pulmonary Disease	24 (12%)
Previous or current pulmonary tuberculosis	57 (28%)
HIV infection (*134 tested)	47 (35%)
Chronic exposure to Biomass Fuel	66 (32%)
Smokers	26 (12%)
**Symptoms & Signs**	
Dyspnea	194 (93%)
Cyanosis	26 (12%)
Fatigue	184 (88%)
Palpitations	153 (73%)
WHO Functional Class III	92 (44%)
WHO Functional Class IV	46 (22%)
6-minute Walking Test <300m	71 (34%)
Right Heart Failure	78 (37%)
Raised Jugular Venous Pressure or Peripheral Edema	174 (83%)
Systolic murmur	119 (57%)

The PAPUCO registry showed major gaps in usage of evidence-based interventions to prevent diagnose and/or manage PH. There was high prescription rate for loop diuretics and spironolactone, in line with the high percentage of patients with congestive heart failure.

No responsiveness to CCB testing was done in most countries, due to unavailability of cardiac catheterization labs or technical expertise to perform the procedure. There was very low usage of disease modifying agents: sildenafil and high dose calcium channel blockers were essentially prescribed in Group 1 and 3; other drugs approved for primary arterial hypertension were not available. Similarly, we unveiled low usage of beta-blockers and anticoagulants in patients with heart failure and atrial fibrillation, respectively.

This registry concluded that in sub-Saharan Africa PVD derives from a high burden of and interaction between non-communicable and infectious diseases. It affects mostly the young – and in particular females – presenting in advanced stages of HF. Survival is very poor, linked to profound lack of suitable and affordable management options, especially in the context of various etiologies requiring technically challenging diagnostic procedures and open heart surgery for their management. The authors proposed the design of pragmatic management guidelines to improve outcomes, particularly considering that access to right heart catheterization is limited and PH-specific therapies remain largely unaffordable in the region; the Pediatric and Congenital Heart Disease Task Forces of the Pulmonary Vascular Research Institute partially addressed this request in their consensus statements, by providing guidelines for the minimal investigative procedures for repair of congenital heart disease with associated PH in children^24^ and indications for cardiac catheterization in children with pulmonary hypertensive vascular disease^25^.

Similar guidelines with such indications and cut-offs for surgery of multiple rheumatic valve heart disease, management of secondary pulmonary hypertension, management of tuberculosis-related PVD, etc. are lacking and may come from well conceived and implemented registries from endemic places such as the African continent.

We briefly present two case reports (**see boxes**) illustrating how multifactorial PVD can present in Africa, and how important is the role of infectious PVD as a cause of disability and premature death in the region.

Case Report Box 1 **Infectious pulmonary vascular disease: Sequelae of perinatal HIV infection, recurrent pulmonary tuberculosis and drop-out of antiretroviral treatment**

**Figure 1.**
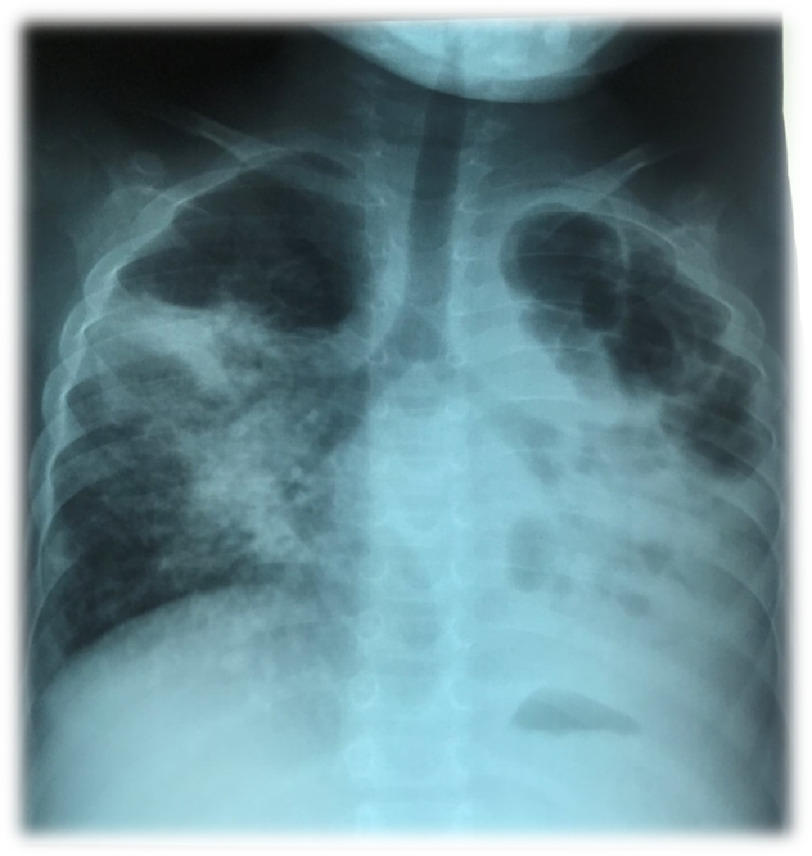
Chest X-ray of a 7-years old girl with perinatal HIV infection, followed by recurrent pulmonary tuberculosis and persistent high viral load associated with poor adherence to therapy for both HIV and tuberculosis. The image shows apical pleural thickening and cardiac silhoute not well defined due to heterogeneous opacities and centrilobular nodular pattern affecting the right medial and basal middle lobe, due to possible transbronchial dissemination from the left lung to the middle lobe of the right lung, suggesting a re-activation of tuberculosis. Left lung “destroyed” as a result of tuberculosis, in the upper lobe there are areas of emphysema and fibrosis, as well as consolidation of the lower lobe with cavitation (cavernous aspect) and bronchiectasis.

A 7-year-old Mozambican girl, daughter of HIV-positive parents, living in a suburb of a poor urban setting in a low-income country, presented to our cardiac clinic brought by her grandmother in September 2018 on NYHA functional class III-IV with a history of ten weeks of fever, productive cough and grade III dyspnea. On physical examination she presented tachypnea, regular tachycardia at 108/minute, intercostal drawing and mild peripheral hypoxia (SPO2 92%) at rest on ambient air. She also had peripheral cyanosis, clubbed fingers, lower abdominal distension, hepatomegaly (three cm below the costal border) and lower extremity edema. The chest auscultation revealed absence of palpable heart impulse, systolic murmur 3/6 over the tricuspid area, apical tubal murmur, abundant bilateral bullous snores in the lung fields and discrete basal crepitations. The HIV test performed at that time was inconclusive/undetermined, with a CD4 count of 197 cells/mm^3^ (corresponding to 13%). Other relevant exams revealed a white blood cell count of 13 × 10^3^, hypochromic and mycrocitic anemia (hemoglobin 7.8 g/dl; MCV 67, CMHC 21); normal renal and liver function. No ECG was available and the chest X-ray (Figure 1) revealed severe lung parenchyma destruction, cavernous aspect large cavities in the apical regions, heterogeneous opacities and presence of centrilobular nodules, and bronchiectasis. The transthoracic cardiac ultrasound revealed marked hypertrophy and dilatation of the right ventricle with preserved systolic function, dilatation of the right atrium without any evidence of thrombi, normal pulmonary valve, dilatation of the pulmonary arteries and severely elevated pulmonary pressures – as estimated by velocity gradient across the tricuspid valve regurgitation, at 4,7 m/s; the right filling pressure was increased, as measured by the distended inferior vena cava, and the evaluation excluded any congenital heart disease with left-to-right shunt.

The child had been exposed to continuous indoor biomass fuel pollutants from birth, mainly coal and wood, had first TB treatment in 2012, previous respiratory infections and two previous admissions. She had started pulmonary tuberculosis re-treatment and second-line antiretroviral therapy in October 2017 (after being non-adherent). According to the grandmother, the family was facing social and economic issues: the child’s mother had to be home taking care of a 2-year-old HIV-infected sibling who was also sick, while being pregnant of her third child; therefore, the grandmother decided to take the little girl from her parents to help with day-to-day care. The grandmother (also HIV infected but following ART with undetectable viral loads) was motivated to take care of her granddaughter, but the parents wanted the child back.

The diagnosis of cor pulmonale associated with chronic obstructive pulmonary disease caused by recurrent pulmonary tuberculosis (and possibly other recurrent pulmonary infections) was considered. The girl was treated with diuretics (furosemide and potassium sparing amiloryde) in addition to antiretroviral therapy, antibiotics, prophylaxis for pneumocystis (jirovecii) pneumonia with cotrimoxazole, and correction of anemia with ferrous salt and folic acid. She failed to present to the following appointment, and after contacting the grandmother via cellphone we learned that she had died in January 2019.

Case Report Box 2 **Multifactorial pulmonary hypertension: Left endomyocardial fibrosis with severe pulmonary hypertension and active schistosomiasis affecting multiple organs**

We have previously reported on a 13-year-old Mozambican boy presented to Maputo Central Hospital with right diastolic cardiac failure, referred from an high endemic zone of endomyocardial fibrosis (EMF)^26^. In summary, the boy reported a 3-month history of progressive exertional dyspnea, chest pain exacerbated by exercise, and abdominal distension. He reported no past medical history and no family history of cardiac problems. On examination he was apyretic with a heart rate of 108 bpm, regular, blood pressure of 90/60 mmHg, respiratory rate of 16 breaths per minute. Cardiac examination revealed a raised jugular venous pressure with prominent CV wave, a visible pulsating, palpable, non-displaced apex beat, and a mild holo-systolic murmur on auscultation. A three cm hepatomegaly was observed, but no pedal edema was present, as is usually the case in children with EMF.

HIV and malaria tests were negative; erythrosedimentation rate was at 55 mm/h; leucocytes at 16.9 × 10^3^/μL with 40.6% of eosinophil’s. The chest X-ray showed increased cardio-thoracic rate due to enlargement of atria, and pulmonary congestion. On the electrocardiogram there was sinus tachycardia, signs of bi-atrial enlargement and features of important left sub-endocardial lesion. The echocardiography revealed a homogeneous mass like a thrombus occupying the apex of the left ventricle, a small right ventricle due to amputation of the apex by extensive fibrosis, large dilatation of both atria, and severe tricuspid regurgitation allowing the estimation of systolic pulmonary pressure at nearly the systemic values.

**Figure 2.**
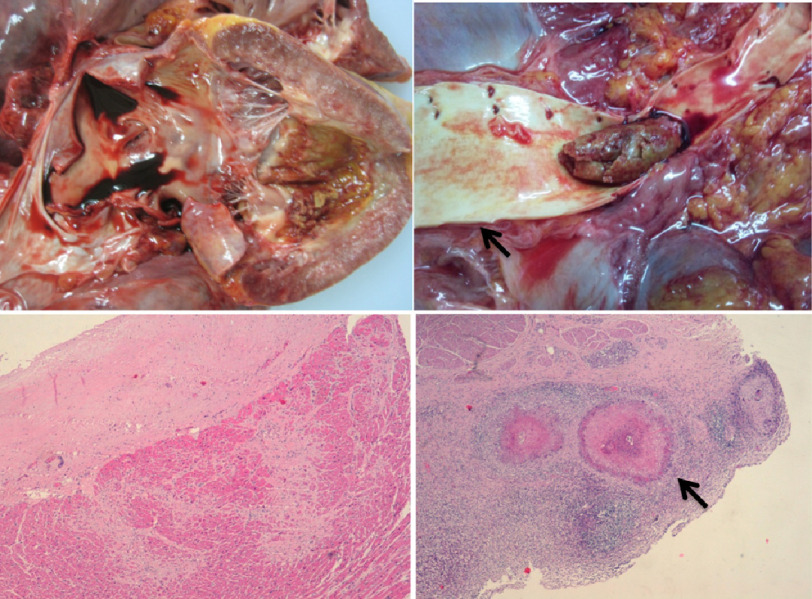
Left ventricle anfractuous surface (upper left, arrow) in an area that corresponds to the basis of a thrombus previously detached to the aorta (upper right). Hematoxylin-Eosin 100x right ventricular endomyocardial sample revealing marked hyaline thickness of endocardium, scarce cellularity and large fibrosis bands into the myocardium (lower left). Eosinophilic granulomas centered by viable Schistossoma eggs (lower right, arrow) in a sample of the urinary bladder.

Due to his severe condition and in the absence of consensus regarding the indication for surgery immediate surgery the patient was admitted and treated with diuretics, but despite the resolution of the dyspnea he died suddenly on day 6 after admission. On autopsy there was an enlarged hearth due to bi-atrial dilatation, the RV was amputated by fibrosis with marked retraction of the apex and the endocardium and inner myocardium of the LV thickened with fibrosis. The surface of the LV apex was anfractuous, in an area corresponding to the base of a thrombus that had detached and embolized to the abdominal aorta. Histological studies with Hematoxylin-Eosin, Van Gieson, and Masson Trichromic stains in cardiac samples revealed right ventricle endocardial thickening by dense fibrosis with hyalinization, large fibrosis bands penetrating to the myocardium, neovascularization and scarce chronic inflammatory infiltrated. The same features were seen in the LV, but the neovascularization and the chronic inflammatory infiltrate was more intense; in addition, there was fibrin deposits in the thrombus detachment zone. Eosinophilic granulomas were found in the myocardium, liver and bladder. Additionally, there was chronic passive congestion of the liver and spleen; pulmonary chronic congestion; hepatic periportal fibrosis associated with the presence of eosinophilic granulomas; and active polipoyd cystitis with eosinophilic granulomas centered by Schistosoma eggs (Figure 2).

This case of concomitant active schistosomiasis and chronic endomyocardial fibrosis with major diastolic dysfunction that leads to severe pulmonary hypertension represents another example of multifactorial pulmonary vascular disease in Africa.

## Discussion and lessons learned

**Figure 3.**
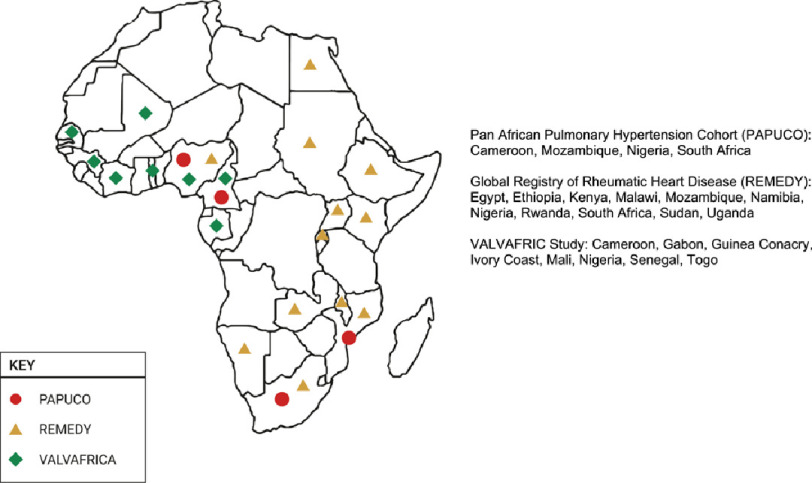
Countries participating in the multinational registries in Africa that assessed the occurrence of pulmonary vascular disease. PAPUCO is, to date, the only all Africa disease specific registry for pulmonary hypertension, while REMEDY and VALVAFRIC were both for rheumatic heart disease, with the latter involving exclusively African countries.

There are only three multinational registries of cardiovascular diseases dedicated exclusively to African patients from which data on the occurrence of PH could be obtained^3, 7, 8^. Among the lessons learned with the implementation of these registries – and in particular from the only registry dedicated to pulmonary hypertension, the PAPUCO study - is the diverse etiology of PVD in Africa. Although not covering all countries in the continent (Figure 3), these registries have uncovered some unique features of PVD in the region. Their results suggest that, at least partially, this high occurrence of interstitial and vascular pulmonary disease that results in PH is related to; chronic exposure to indoor air pollution, multiple respiratory illnesses including tuberculosis, high prevalence of schistosomiasis and HIV, as well as other poverty-related environmental and/or occupational hazards.

**Case 1:** Mozambique has a high prevalence of HIV, especially in the adolescent population aged between 15 and 19 years; the prevalence is around 2.5% in boys and 6.2% in girls. Moreover, HIV infection is a major cause of death for those aged between 15 and 24 years.^26^ The ambitious goal of reaching HIV treatment goals by 90–90–90 by 2020 – ie 90% of people living with HIV know they are HIV positive, 90% of people who know they are HIV positive are 90% of people on ART have viral suppression^27^ – was set in Mozambique, and progress towards these goals has been measured through the IMASIDA surveys^28^. The results of the 2015 cycle were:

i. 40% of women and men aged 15–49 living with HIV knew their HIV status;
ii. 86% of women and men aged 15–49 who knew their HIV status were on ART;
iii. 68% of women and men aged 15–49 who took ART had suppressed viral load^28^.

HIV testing for pregnant women, a key step in reducing mother-to-child transmission of HIV usually performed during antenatal visits, increased from 44% in IMASIDA 2009 to 67% in IMASIDA 2015^28^. Only 44% of HIV-positive and pregnant/lactating women started ART, and 2% of 6–23 month olds are HIV positive, prior to systematic testing of pregnant women and prevention of maternal to child transmission in Mozambique.

The severe and fatal pulmonary vascular disease in the child we presented resulted from multiple factors namely chronic exposure to biomass fuel, perinatal HIV infection, superimposed recidicant pulmonary tuberculosis and recurrent interstitial pneumonia, all known to be risk factors for pulmonary hypertension^29–32^.

Low health literacy of parents/carers, weak social support, and poor adherence to therapy explain the recurrence of tuberculosis. Poor control of HIV infection and severe pulmonary complications early in life, resulting in severe right heart failure, major disability and premature mortality.

**Case 2:** EMF, a restrictive cardiomyopathy of unclear etiology that affects children and young adults, with high prevalence in some parts of sub-Saharan Africa^33^, is a major cause of disability in those affected. PH is in fact the commonest cause of death in patients with severe left EMF^34^, due to both left ventricular diastolic dysfunction; chronic thromboembolism is a second mechanism related to detachment of emboli from thrombus inside an aneurismal right atrium. The patient we present had also schistosomiasis, still a common cause of PVD in Africa^35^, despite mass and targeted administration of praziquantel to schoolchildren and adults in many countries^36–38^.

These cases underline the need not only for major investments in prevention of environmental and infectious dterminants of PVD in Africa, but also improvement in health care and social support for those with lower socioeconomic background.

They stress the need for an equity agenda to address the stage of complications, including universal health coverage to ensure continuous specialized care dor chronic condition such as PH. Moreover, they illustrate the challenges for the management of PVD in under-resourced settings in Africa where uncontrolled infections coexist with poverty-related non-communicable diseases.

Finally, they stress the need for prevention and early detection to prevent adverse outcomes, as well as registries to explore the potential synergistic effects of prevalent risk factors such as chronic exposure to biomass fuel and infectious agents in determining cardiopulmonary disease in general, and severe pulmonary vascular disease in particular. Such registries may help understanding the natural history and progression of PVD caused by such endemic conditions, thus allowing the identification of new therapeutic targets and better outcomes.

## Perspectives

The unacceptably high burden of PVD in Africa highlights the need for the development of affordable and scalable algorithms for early diagnosis and adequate management of those detected in underserved areas. Recently, the concept of cardiopulmonary disease (CPD) as a broad spectrum of conditions concurrently affecting the heart and lungs has been suggested. This entity ranges from people at “high risk” of developing RHF and mortality due to the presence of largely asymptomatic mild-to-moderate elevation of pulmonary pressures, to those who have already developed concurrent and symptomatic lung/cardiac pathology^39^.

Common left heart diseases with reduced ventricular ejection fraction (dilated cardiomyopathy, peripartum cardiomyopathy, ischaemic heart disease), left ventricular diastolic dysfunction (severe uncontrolled systemic hypertension) or abnormal valve function (rheumatic heart disease) often result in pulmonary oedema and subsequent poor lung compliance. Additionally, multifactorial CPD can also occur in some regions in Africa due to high occurrence of pulmonary tuberculosis, sickle cell disease and particular conditions such as endomyocardial fibrosis. Finally, the HIV epidemic has triggered new pathways to CPD via immunosuppression and opportunistic infections, cardiac dysfunction linked to HIV-related cardiomyopathy, transplacental exposure to drugs like zidovudine and primary PH in those untreated.^40^ This said, CPD remains an under-appreciated contributor to poor health outcomes due to a shortage of human and material resources to screen for and diagnose this complex entity.

With this new knowledge Africa’s record of diagnosing severe PH in patients already in functional classes III and IV of the NYHA at the few specialized centres can now be challenged. Newly-identified thresholds of pulmonary pressure elevation that correspond to high-risk for premature mortality, associated with often silent and untreated PH,^41^ support the idea of combining early detection of at risk patients and long-term follow up to clarify the observed pattern of CPD and high incidence of RHF in Africa. Pragmatic screening protocols for identifying early stages of CPD have already been proposed for use in these contexts, aiming at allowing prevention and early recognition of PVD and/or people at risk^39, 42^.

The growth in use of information and communication technology in the continent has allowed the some registries to be performed using web-based databases^3, 9^. In parallel, pioneer studies were performed in Africa using ultrasound for community-based research on neglected cardiovascular diseases in low-income settings^43, 44^. Moreover, the addition of rapid testing and point-of-care devices for these community-based research efforts is currently possible in poor settings. This experience and the constant progress in technology and knowledge allow us to foresee in the near future research into the incidence, prevalence and natural history of PVD in Africa.

Existing networks of experts and researchers in the cardiovascular and pulmonary fields - such as the Pan African Society of Cardiology (PASCAR), the Non-Communicable Diseases and Injury of the Poorest Billion (NCDI Poverty), the Global Alliance for Respiratory Diseases (GARD) and the Pulmonary Vascular Research Institute (PVRI) – may link professionals geographically apart in joint efforts not only to define the profile of PVD in the region, but also to explore geographic, environmental and seasonal differentials that may inform policy and care provision within various regions of the continent. Particularly for PVD registries, PVRI may act as a promoter of high quality research using pragmatic clinical and ultrasound diagnostic algorithms, coupled with point-of-care detection of biomarkers of current and past infections, as well as of asymptomatic heart and respiratory failure.
